# Assessment of tolerability and acceptability of an alcohol-based hand rub according to a WHO protocol and using apparatus tests

**DOI:** 10.1186/s13756-019-0646-8

**Published:** 2019-11-27

**Authors:** Patryk Tarka, Katarzyna Gutkowska, Aneta Nitsch-Osuch

**Affiliations:** Department of Social Medicine and Public Health, Medical University of Warsaw, ul Oczki 3, 02-007 Warsaw, Poland

**Keywords:** Alcohol-based hand rub, Hand hygiene, WHO protocol, Tolerability, Acceptability

## Abstract

**Background:**

The effectiveness of alcohol-based hand rubs (ABHRs) depends substantially on their acceptability and tolerability. In this study, we assessed the acceptability and tolerability of a new ABHR (product EU 100.2018.02).

**Methods:**

Among physicians, nurses, and cosmetologists who used the ABHR for 30 days, we assessed the product’s acceptability and tolerability according to a WHO protocol. Additionally, we used instrumental skin tests. Participants assessed the product’s color, smell, texture, irritation, drying effect, ease of use, speed of drying, and application, and they gave an overall evaluation. Moreover, they rated the tolerability, i.e. their skin condition, on the following dimensions: intactness, moisture content, sensation, and integrity of the skin. The tolerability was also assessed by an observer as follows: redness, scaliness, fissures, and overall score for the skin condition. Instrumental skin tests included transepidermal water loss, skin hydration, sebum secretion, and percentage of skin affected by discolorations. All assessments were made at baseline (visit 1), and 3–5 days (visit 2) and 30 days (visit 3) later.

**Results:**

We enrolled 126 participants (110 [87%] women) with a mean age of 34.3 ± 11.65 years. Sixty-five participants (52%) were healthcare professionals (physicians, nurses), and 61 (48%) were cosmetologists. During visit 2 and visit 3, about 90% of participants gave responses complying with the WHO’s benchmark for acceptability and tolerability. Similarly, the ABHR met the WHO criteria for observer-assessed tolerability: on all visits, in more than 95% of participants, the observer gave scores complying with the WHO benchmark. Transepidermal water loss decreased from baseline to visit 3 (*p* < 0.001), whereas skin hydration, sebum secretion, and the percentage of skin affected by discolorations did not change significantly during the study (*p* ≥ 130).

**Conclusions:**

The EU 100.2018.02 had both high acceptability and tolerability, meeting the WHO criteria. The WHO protocol proved useful in the analysis of acceptability and tolerability of ABHRs.

## Background

Hand hygiene plays a central role in the prevention of infections, including those with multidrug-resistant pathogens [1, 2]. However, in healthcare and cosmetology, hand hygiene is insufficient, which is associated with increased morbidity, mortality, and healthcare costs [3, 4].

Although consistent use of alcohol-based hand rubs (ABHR) improves hand hygiene substantially, poor acceptability and tolerability of ABHRs in the workplace is one of the most common causes of ineffective hand hygiene [5, 6]. Thus, acceptability and tolerability are important criteria for selecting ABHRs, and high acceptability and tolerability help maintain hand hygiene practices [7–9]. In 2009, the WHO put forward both a protocol and criteria for assessing the acceptability and tolerability of ABHRs [10].

In this study, we evaluated the acceptability and tolerability of a new ABHR (product EU 100.2018.02) among healthcare professionals and cosmetologists. We followed the WHO protocol and used additional instrumental skin tests.

## Methods

### Participants

We enrolled adult participants (> 18 years) among physicians and nurses of a gynecological ward and among cosmetologists of a private clinic. We excluded people with skin diseases or known hypersensitivities to the ingredients of the investigational product. The study was approved by the Bioethics Committee of the Medical University of Warsaw (KB213/2018). All participants signed informed consent before enrollment. Basic characteristics of participants were gathered in accordance with the WHO protocol [10].

### Investigational product

The investigational product, an ABHR (EU 100.2018.02, HCS Europe, Poland), complied with the EN 1500:2013–07 and EN 12791 + A1:2017–12 standards. The ABHR contained ethanol CAS 64–17-5 (80 g/100 g), vegetable glycerin, vitamin E, bisabolol, and flavonoids.

### Study procedures

The study assessed the tolerability and acceptability of the investigational product according to the protocol proposed by the WHO [10]. Briefly, on a seven-point Likert scale, participants rated the product’s:

color (“unpleasant”-“pleasant”),

smell (“unpleasant”-“pleasant”),

texture (“sticky”-“non-sticky),

irritation (“very irritating”-“not irritating”),

drying effect (“very much”-“not at all”),

ease of use (“very difficult”-“very easy”),

speed of drying (“very slow”-“very fast”),

application (“unpleasant”-“pleasant”), and.

overall evaluation (“dissatisfied”-“satisfied”).

Similarly, on a seven-point Likert scale, participants assessed the skin condition of their hands:

- appearance (“abnormal”-“normal”);
- intactness (“abnormal”-“normal”);
- moisture content (“abnormal”-“normal”);
- sensation (“abnormal”-“normal”);
- overall integrity of the skin (“very altered”-“not altered”).

Skin condition was also rated by an observer, as follows:

- redness (0–4, no redness-very bright with edema),
- scaliness (0–3, no scaliness-very pronounced separation from skin),
- fissures (0–3, no fissure-extensive cracks with bleeding or seeping), overall
- overall score for the skin condition (0, no observable scale or irritation of any kind; 1, occasional scale that is not necessarily uniformly distributed; 2, dry skin and/or redness; 3, very dry skin with whitish appearance, rough to touch, and/or redness, but without fissures; 4, cracked skin surface but without bleeding/seeping; 5, extensive cracking of skin surface with bleeding/seeping).

All evaluations were carried out at baseline and 3–5 and 30 days after using the ABHR. All participants used 30 ml or more of the ABHR daily. Each participant received a personal container of the ABHR. Participants were allowed to use hand lotion or hand cream throughout the study.

### Instrumental skin assessments

During all three visits, the skin on hands was assessed with an MPA-5 Corneometer to evaluate skin hydration; an MPA-5 Sebumeter to evaluate sebum secretion; a TM300 Tewameter with a cylindrical probe to evaluate transepidermal water loss; and a Derma Visualizer camera to assess the percentage of skin affected by discolorations (all devices by Courage & Khazaka, Cologne, Germany). The Corneometer measures hydration in the stratum corneum of the epidermis based on the electrical properties of the skin; skin hydration is reported in units from 0 to 130 (each unit indicates 0.02 mg of water per square centimeter of the stratum corneum). The Sebumeter measures sebum content in a foil attached previously to the skin for 30 s; the results range from 0 to 350 micrograms of sebum per square centimeter of skin. The Tewameter estimates transepithelial water loss based on the skin’s wetness and temperature, which are used to calculate water vapor pressure. Transepithelial water loss is reported in g/m^2^/h, with higher values indicating worse skin hydration. The Derma Skin Visualizer uses parallel and crossed polarized light to measure pigment discoloration on the skin; the result is given as the percentage of the studied area with discolorations.

### End points

The WHO criteria for product acceptability were as follows:

- ≥ 50% of participants responding above 4 for “Color” and “Smell”, and ≥ 75% of participants responding above 4 for “Texture”, “Irritation”, “Drying effect”, “Ease of use”, “Speed of drying”, “Application”, and “Overall evaluation”.

The WHO criteria for skin tolerability were as follows:

- ≥ 75% of participants responding above 4 for the skin’s “Appearance”, “Intactness”, “Moisture content”, “Sensation”, and “Overall integrity”
- ≥ 75% of participants with scores below 2 on the skin evaluation by the observer.

### Statistical analysis

Variables are presented as counts and percentages or as means±standard deviations (SD). Comparisons were carried out with repeated-measures analysis of variance (ANOVA) and post-hoc Scheffe contrasts. *P* < 0.05 was considered statistically significant. All calculations were carried out with SPSS software (version 2018).

## Results

### Participants

We enrolled 126 participants (110 [87%] women) with a mean age of 34.3 ± 11.65 years. Sixty-five participants (52%) were healthcare professionals (physicians, nurses) and 61 (48%) were cosmetologists. Their mean professional experience was 9 ± 10 years; 88 (67%) participants worked full-time. Thirty-three (26%) participants had non-occupational activates that could damage skin, 10 (15%) participants declared frequent skin irritation, and 88 (70%) participants declared non-frequent skin irritation. Fifty-nine (48%) participants used ABHRs for five years or longer. Forty-five (36%) participants had Fitzpatrick skin type 1, 58 (46%) had Fitzpatrick skin type 2, and 23 (18%) had Fitzpatrick skin type 3. Table 1 shows the frequency of hand hygiene practices assessed during visit 3.

**Table 1:** Frequency of hand hygiene practices assessed at visit 3

N (%)	
*Do you usually use a hand lotion?*	
As often as possible	14 (11.1)
Sometimes, depending on the season	30 (23.8)
Several times daily	33 (26.2)
Once daily	27 (21.4)
Seldom	20 (15.9)
Never	2 (1.6)
*Do you think that a lack of time has an effect on hand hygiene?*	
Always	23 (18.4)
Very often	30 (24.0)
Often	52 (41.6)
Do not know	5 (4.0)
Seldom	6 (4.8)
Very seldom	7 (5.6)
Never	2 (1.6)
*Do you think that skin damage has an effect on hand hygiene?*	
Always	25 (19.8)
Very often	34 (27.0)
Often	47 (37.3)
Do not know	8 (6.3)
Seldom	8 (6.3)
Very seldom	2 (1.6)
Never	2 (1.6)
*During how many consecutive days have you used the test product? (days)*	
3 days	74 (58.7)
4 days	16 (12.7)
5 days	24 (19.0)
6 days	1 (0.8)
7 days	10 (7.9)
> 7 days	1 (0.8)
*How often do you have direct contact with patients during your working day? (contacts)*	
< 1	7 (5.6)
1–5	29 (23.)
6–10	36 (28.6)
11–15	29 (23.0)
> 15	25 (19.8)
*Are there differences between the test product and the product used in your hospital?*	
No	1 (0.8)
I do not think so	1 (0.8)
Do not know	4 (3.2)
I think so	61 (48.4)
Yes	40 (31.7)
Yes, absolutely	19 (15.1)
*Do you think that the investigated product could improve your hand hygiene?*	
No	4 (3.2)
Do not know	8 (6.3)
I think so	60 (47.6)
Yes	21 (16.7)
Yes, absolutely	33 (26.2)

### Self-assessed acceptability and tolerability of the product

**Fig. 1:**
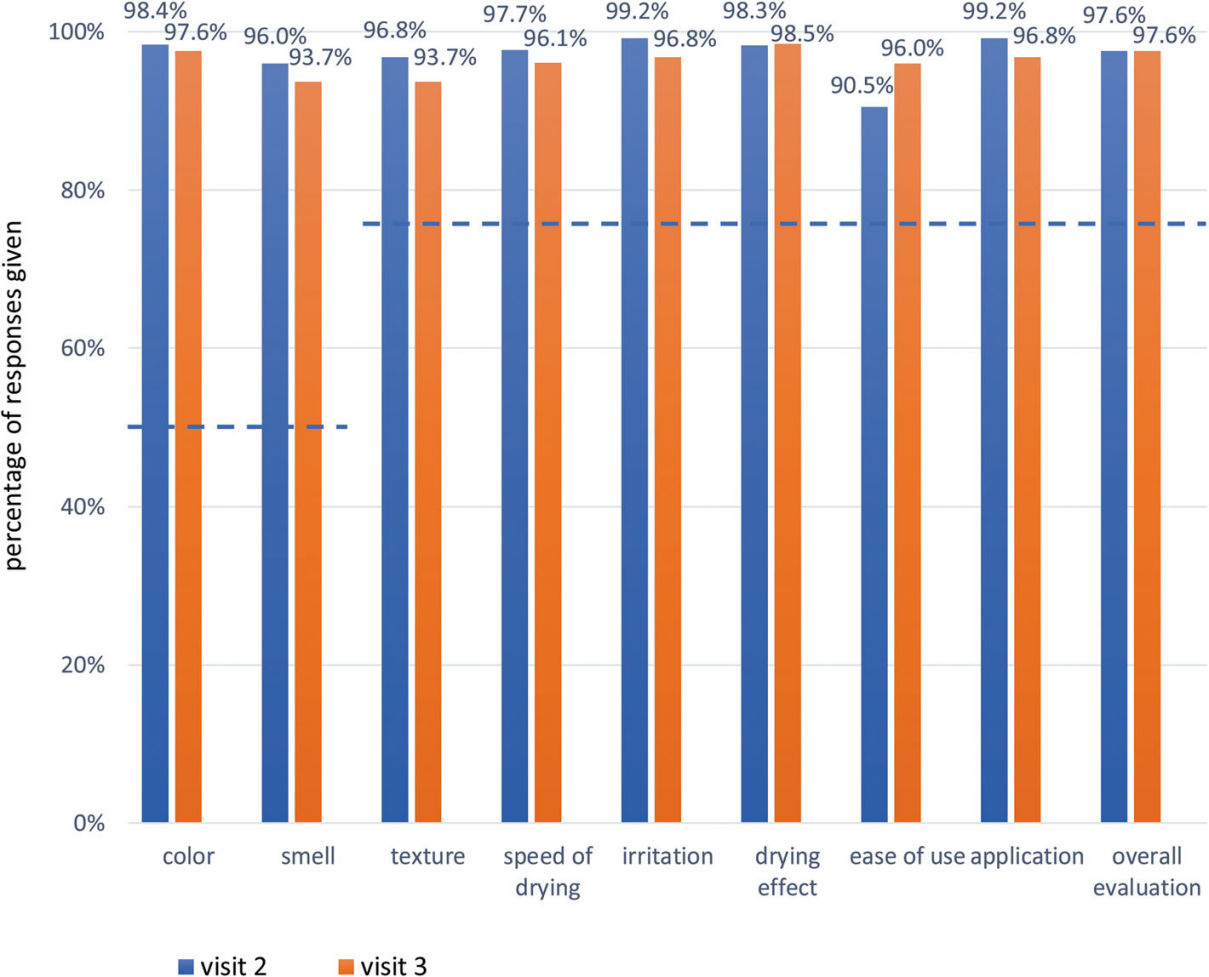
Percentage of participants with responses of 4 or more to questions about the ABHR acceptability. Horizontal lines represent WHO criteria for acceptability

**Fig. 2:**
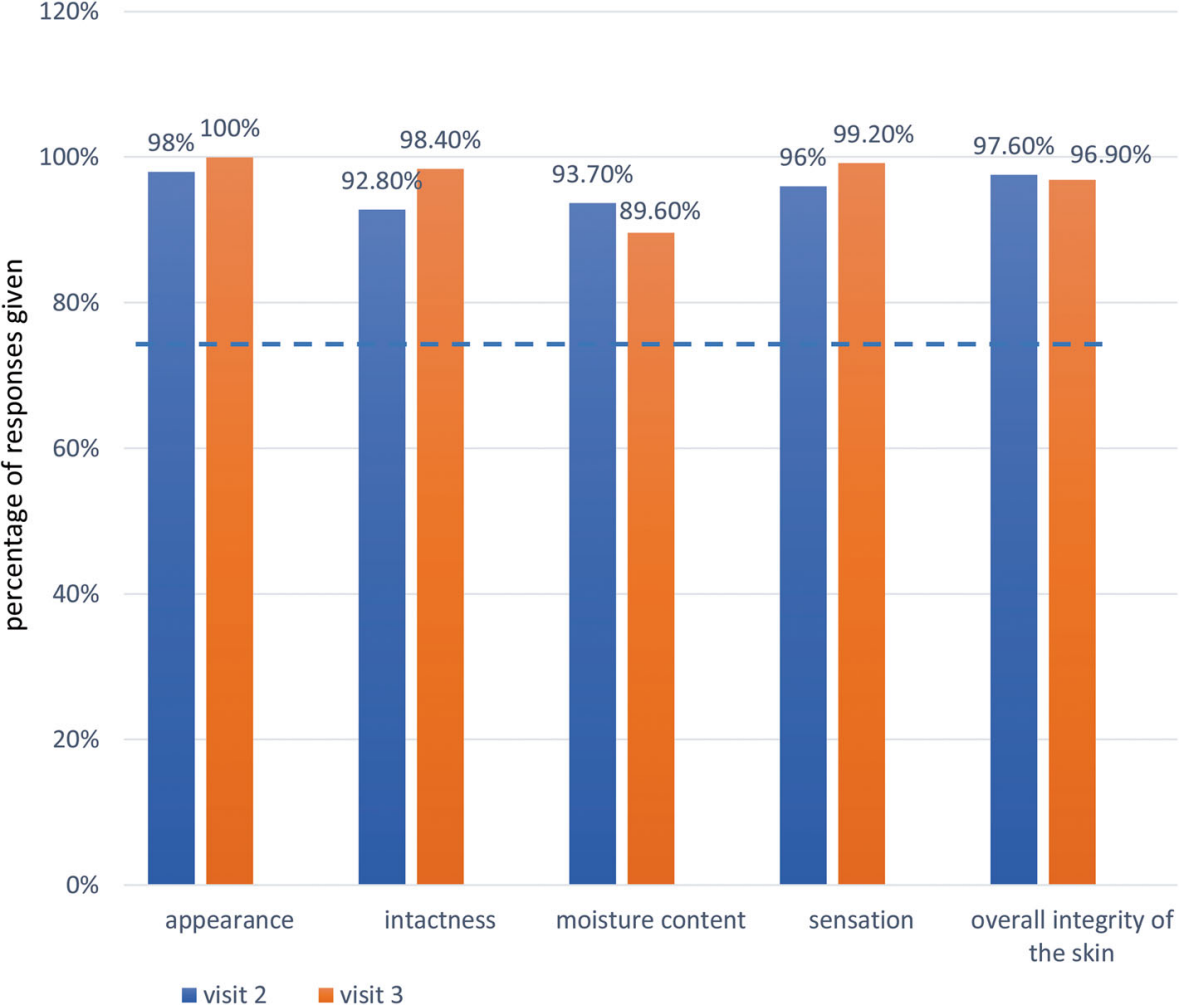
Percentage of participants with responses of 4 or more to questions about skin tolerability. Horizontal line represents WHO criterion for acceptability

During visit 2 and visit 3, the ABHR met the WHO criteria for self-assessed acceptability. More than 90% of participants gave responses of 4 or more to all questions about the product’s acceptability (Fig. 1). Similarly, the ABHR met the WHO criteria for self-assessed tolerability. About 90% or more participants gave responses of 4 or more to all questions about skin condition (Fig. 2).

### Observer-assessed tolerability of the ABHR

The ABHR met the WHO criteria for observer-assessed tolerability. On all visits, in more than 95% of participants, the observer gave scores of ≤1 to all items that assessed skin tolerability (Table 2).

**Table 2:** Assessments of the ABHR tolerability by an observer

Item	Skin condition score	Visit 1	Visit 2	Visit 3
Redness		**N (%)**	**N (%)**	**N (%)**
	No redness (0)	86 (68.3)	96 (76.2)	96 (76.2)
	Slight redness or blotchy (1)	36 (28.6)	26 (20.6)	26 (20.6)
	Moderate redness (2)	2 (1.6)	2 (1.6)	2 (1.6)
	Strong redness (3)	0 (0.0)	0 (0.0)	0 (0.0)
	Fiery red with edema (4)	2 (1.6)	2 (1.6)	2 (1.6)
	Proportion with score < 2*	122 (96.8)	122 (96.8)	122 (96.8)
Scaliness				
	No scales (0)	93 (73.8)	103 (81.7)	103 (81.7)
	Very slight sporadic scales (1)	30 (23.8)	19 (15.1)	19 (15.1)
	Moderate scales (2)	3 (2.4)	4 (3.2)	4 (3.2)
	Considerable scales (3)	0 (0.0)	0 (0.0)	0 (0.0)
	Proportion with score < 2*	123 (97.6)	122 (96.8)	122 (96.8)
Fissures	N			
	No fissures (0)	84 (66.7)	95 (75.4)	97 (77.0)
	Very fine fissures (1)	39 (31.0)	29 (23.0)	26 (20.6)
	Broad sporadic or several fissures (2)	2 (1.6)	2 (1.6)	2 (1.6)
	Widespread cracks with hemorrhage or exudate (3)	1 (0.8)	0 (0.0)	1 (0.8)
	Proportion with score < 2*	123 (97.7)	124 (98.4)	123 (97.6)
Global score	N			
	No dry skin or irritations (0)	105 (83.3)	104 (83.2)	107 (84.9)
	Incidental dry skin (1)	18 (14.3)	17 (13.6)	15 (11.9)
	Dry skin and/or redness (2)	1 (0.8)	2 (1.6)	3 (2.4)
	Very dry whitish rough skin (3)	2 (1.6)	1 (0.8)	1 (0.8)
	Chapped skin without hemorrhage or exudate (4)	0 (0.0)	0 (0.0)	0 (0.0)
	Widespread fissures with hemorrhage or exudate (5)	0 (0.0)	1 (0.8)	0 (0.0)
	Proportion with score < 2*	123 (97.6)	121 (96.8)	122 (96.8)

* WHO criterion for tolerability

### Instrumental skin assessments

**Fig. 3:**
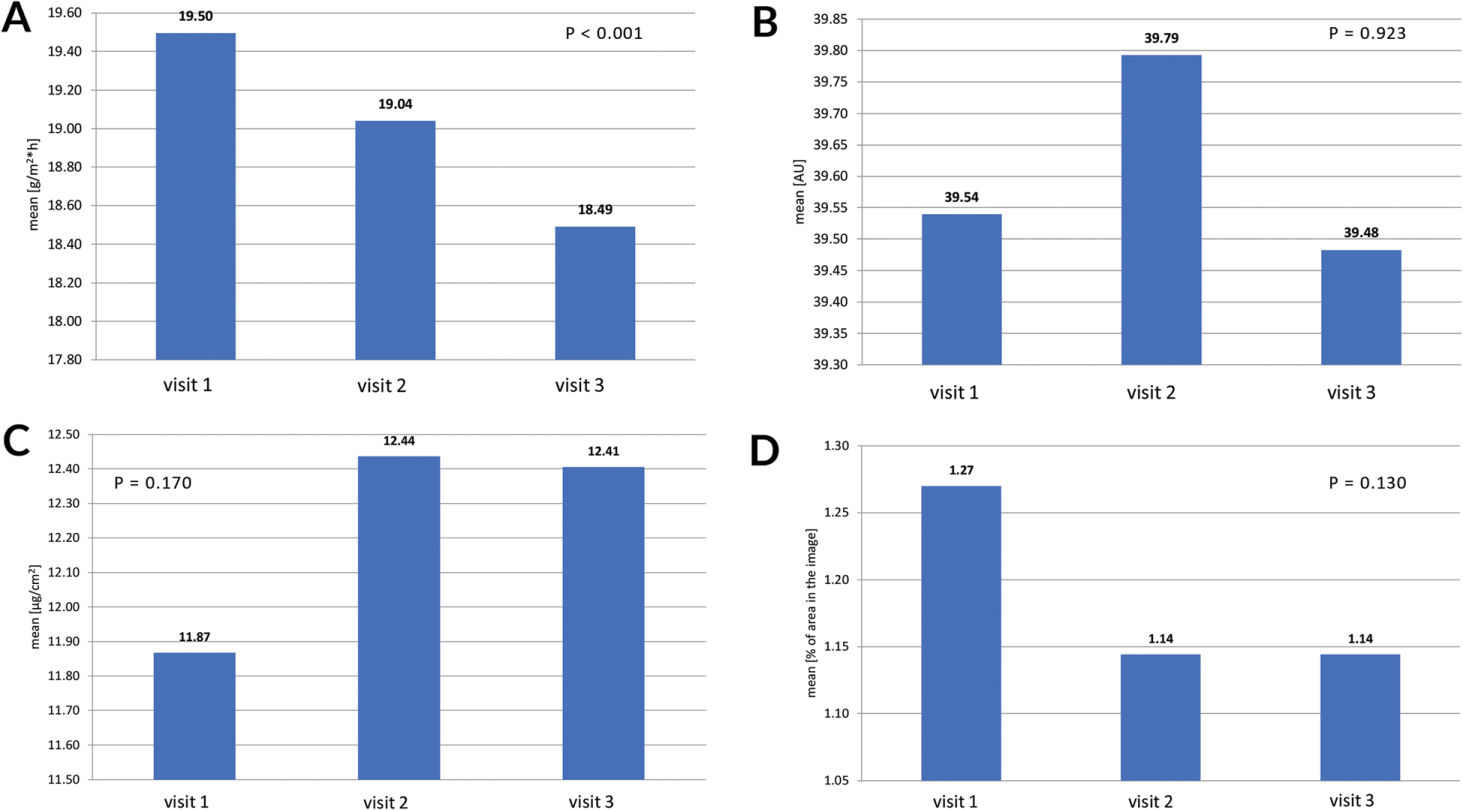
Instrumental skin assessments. A. Transepidermal water loss, B. Skin hydration, C. Sebum secretion, D. Percentage of skin affected by discolorations

Transepidermal water loss decreased from baseline to visit 3 (*p* < 0.001); only the difference between visit 1 and visit 3 was significant on post-hoc comparisons (*p* < 0.05, Fig. 3 A). Skin hydration, sebum secretion, and number of discolorations on the skin on hands did not change significantly during the study (*p* ≥ 130, Fig. 3 B-D).

## Discussion

This study showed that the investigational ABHR (EU 100.2018.02) had good acceptability and tolerability among healthcare professionals and cosmetologists. The ABHR met all WHO criteria for both acceptability and tolerability. Moreover, participants declared that the product could improve hand hygiene in their workplace.

Hand hygiene is insufficient among healthcare professionals and other staff [1]. For example, Anwar et al. found that hand hygiene is observed only 30% of the time [11]. Lack of time is one of the reasons for poor hand hygiene, which was observed among our participants and in previous studies [12, 13]. Over 80% of our participants admitted that a lack of time causes non-compliance with hand hygiene procedures.

Acceptability and tolerability of hand sanitizer products are also very important for consistent hand hygiene. Therefore, improvements in acceptability and tolerability can translate into fewer infections. Hand products, particularly those containing sodium laureth sulfates, irritate the skin and deplete skin lipids [14–16]. ABHRs seem to be better tolerated than detergents and are now recommended by the WHO as preferred hand sanitizers [17–19]. Because the skin tolerates ethanol better than n-propanol or isopropanol, ethanol is the preferred active substance in ABHRs (also included in the product tested in our study) [20, 21]. Additionally, ethanol has a potent killing effect against viruses [22]. Emollients or other skin conditioners can substantially reduce the skin drying effect of alcohol [23–26]. The investigational product in our study contained vegetable glycerin as a skin conditioner. Importantly, in developmental studies, glycerin did not reduce the bactericidal effect of the product. Moreover, bisabolol, another ingredient of the tested ABHR, has a soothing effect on the skin, in addition to its anti-inflammatory and antibacterial effects [27].

Because ABHRs are important for hygiene, the WHO put forward both a protocol and criteria for assessing the acceptability and tolerability of these products. To our knowledge, only one published study followed this protocol. In that study, in contrast to our study, the product investigated did not meet all the WHO criteria for acceptability [28]. In the study by Wolsfensbergeret et al., the ABHR was too sticky, and it dried too slowly [28]. In contrast, in our study, more than 90% of participants were satisfied with the ABHR’s texture and speed of drying. Moreover, in more than 95% of participants in our study, the product’s tolerability complied with the WHO benchmark. We did not observe any adverse effects of the ABHR, although such products could induce, for example, contact dermatitis, phototoxicity, or pruritus.

We found that the ABHR significantly reduced transepidermal water loss over a month of use, although previous studies on ABHRs found no such effect [29, 30]. Moreover, the ABHR did not impair skin hydration or sebum secretion, nor did it induce skin discolorations. These findings are in line with the excellent acceptability and tolerability of the tested product reported by participants and the observer. However, one has to keep in mind that the study lasted for only one month, and skin condition could change over a longer period. Although the instrumental skin test enabled an objective assessment of skin condition, we needed twice as much time to complete all assessments compared to the WHO protocol alone.

In our study, each participant received a personal container of the tested ABHR, which could have had an effect of the perceived acceptability and tolerability. Wolsfensbergeret et al. found that hand sanitizers available from personal containers might improve self-reported assessments [28]. Participants in our study were allowed to used hand lotions or creams, which could have interfered with our assessments. Moreover, the study was carried out in the winter, which could have worsened the condition of the skin.

## Conclusions

In conclusion, we found that the investigational ABHR (EU 100.2018.02) had high acceptability and tolerability. Thus, it could improve hand hygiene. The WHO protocol proved to be a useful tool in the analysis of acceptability and tolerability of ABHRs.

## Data Availability

The datasets used and/or analyzed during the current study are available from the corresponding author on reasonable request.

## Abbreviations

ABHR: alcohol-based hand rub
EU: European Union
WHO: World Health Organization
